# Design and Analysis of Optical–Mechanical–Thermal Systems for a High-Resolution Space Camera

**DOI:** 10.3390/s25247617

**Published:** 2025-12-16

**Authors:** Xiaohan Liu, Jian Jiao, Kaihui Gu, Hong Li, Wenying Zhang, Siqi Zhang, Wei Zhao, Zhaohui Pei, Bo Zhang, Zhifeng Cheng, Feng Yang

**Affiliations:** 1 Jilin Engineering Laboratory for Quantum Information Technology, Jilin Engineering Normal University, Changchun 130052, China; guba510@126.com (K.G.); lihong@jlenu.edu.cn (H.L.); siqizhang88@163.com (S.Z.);; 2 Institute of Quantum Science and Technology, Yanbian University, Yanji 133002, China; 3 School of Physics, Changchun Normal University, Changchun 130032, China; xinhe7hl@126.com; 4 Changchun UP Optotech (Holding) Co., Ltd., Changchun 130031, China; mengyu409cu@163.com (Z.P.); valen888_81@163.com (B.Z.); 5 Changchun Institute of Optics, Fine Mechanics and Physics, Chinese Academy of Sciences, Changchun 130033, China

**Keywords:** main support structure, finite element simulation analysis, root mean square surface error (RMS), fundamental frequency, primary mirror

## Abstract

To meet the requirements of high resolution, compact size, and ultra-lightweight for micro–nano satellite optoelectronic payloads while ensuring high structural stability during launch and in-orbit operation, mirrors were designed with high surface accuracy. The opto-thermo-mechanical system of the space camera was designed and analyzed accordingly. First, an optical system was designed to achieve high resolution and a compact form factor. A coaxial triple-reflector configuration with multiple refractive paths was adopted, which significantly shortened the optical path and laid the foundation for a lightweight, compact structure. This design also defined the accuracy and tolerance requirements for the primary and secondary mirrors. Subsequently, mathematical models for topology optimization and dimensional optimization were established to optimize the design of the main support structure, primary mirror, and secondary mirror. Two design schemes for the main support structure and primary mirror were compared. Steady-state thermal analysis and thermal control design were carried out for both mirrors. Simulations were then performed on the main system (including the primary/secondary mirror assemblies and the main support structure). Under the combined effects of gravity, a 4 °C temperature increase, and an assembly flatness deviation of 0.01 mm, the surface accuracy of both mirrors, the displacement of the secondary mirror relative to the primary mirror reference, and the tilt angle all met the overall specification requirements. The system’s first-order natural frequency was 156.731 Hz. After precision machining, fabrication, and assembly, wavefront aberration testing was conducted on the main system with the optical axis horizontal. Under gravity, the root mean square (RMS) wavefront error at the center of the field of view was 0.073λ, satisfying the specification of ≤1/14λ. The fundamental frequency measured during vibration testing was 153.09 Hz, which aligned closely with the simulated value and well exceeded the requirement of 100 Hz. Additionally, in-orbit imaging verification was conducted. All results satisfied the technical specifications of the satellite’s overall requirements.

## 1. Introduction

Micro–nano satellites weighing less than 100 kg are characterized by their compact size, light weight, and low launch cost. They have been widely used in fields such as environmental monitoring, geological surveys, and meteorological observation [1,2,3]. The optical payloads onboard these satellites typically provide ground resolutions ranging from 1 to 10 m, fitting within the size and mass constraints of micro–nano satellites. With the advancement of space technology, many applications now demand sub-meter (better than 1 m) or even higher (e.g., 0.5-m) ground resolution to capture finer details. It is increasingly common for a single satellite to carry dual-resolution cameras: one with meter-level resolution for wide-area observation and another with sub-meter resolution for target identification. Achieving very high resolution requires optical systems with longer focal lengths, larger volumes, and greater mass. Consequently, researchers worldwide have conducted extensive studies on how to attain high resolution while simultaneously achieving ultra-lightweight and miniaturized designs. Table 1 summarizes the challenge of achieving sub-meter resolution for microsatellites [4,5,6,7,8,9]. It shows that higher resolution is generally associated with greater mass; even a modest improvement in resolution can lead to a significant increase in weight.

High-resolution cameras for micro–nano satellites face even stricter size and mass constraints, necessitating opto-mechanical systems that are extremely compact and lightweight. These systems must also withstand extreme conditions, including the violent vibrations and shocks during launch, drastic on-orbit temperature variations, and the vacuum of space. To preserve high-resolution imaging quality, relative displacements between optical components and mirror surface deformations must be controlled within minimal allowable tolerances. Worldwide research efforts have therefore focused intensely on achieving high resolution while adhering to the strict size and weight limits of micro–nano satellites. From an optical design perspective, techniques such as Cassegrain multi-folded paths, ultra-compact off-axis three-mirror designs, and freeform optical systems are employed to minimize the camera’s track length, laying the foundation for miniaturization and ultra-lightweight construction. In the domain of mechanical and thermal control design, various innovative approaches have been developed. For instance, Zhou et al. devised a highly thermally adaptive, integrated primary mirror support technology to maintain surface accuracy under thermal loads while achieving an extreme lightweight structure [10]. Li et al. optimized a compact space camera via integrated opto-thermo-mechanical (OTM) analysis, attaining a total mass below 10 kg with system wavefront error better than λ/10 [11]. Lin et al. studied thermal deformation in a telescope’s primary support structure, implementing a passive thermal compensation design using carbon fiber trusses. They established a functional relationship between the coefficients of thermal expansion and truss member lengths to meet specific system requirements [12]. Peng et al. designed a titanium alloy, thin-walled main support structure for a space camera based on Thomason’s cantilever beam theory and an active fitting optimization algorithm. Utilizing selective laser melting (SLM), they fabricated a structure with a natural frequency of 105.97 Hz [13]. Wei et al. applied topology optimization and finite element analysis to enhance the static and dynamic performance of a camera’s opto-mechanical system, achieving ultra-lightweight and high stability [14]. Yin et al. proposed a novel, highly stable, lightweight design for the primary support structure by integrating thin-walled cylinders with support rods [15].

Significant progress has been made in recent years to enhance the resolution of micro–nano satellite cameras within strict volume and mass limits. Advancements span optical design, material selection, OTM system design, and innovative manufacturing processes. For the main support structure, materials like aluminum alloy, titanium alloy, or carbon fiber are typical, often configured in tubular or truss forms [16]. To date, there are no public reports on the use of silicon carbide (SiC) for this component. As the mirror aperture for these cameras is generally below one meter, traditional three-point back support is common, and integrated SiC mirrors remain relatively rare. These emerging technologies represent a promising research direction for developing ultra-lightweight, high-resolution cameras capable of maintaining superior surface accuracy over long-term missions.

This study presents a compact coaxial three-mirror optical system designed for a microsatellite. The system achieves a ground resolution of 0.5 m from a 500 km sun-synchronous orbit and supports both still and video imaging. A key feature is the extensive use of silicon carbide (SiC) for the entire opto-mechanical system, including all mirrors, the main support structure, and the backplate. All mirrors feature an integrated design with lightweight triangular grooves on the back. The primary mirror is connected to the backplate via three flexible hinges. Innovatively, the main support structure adopts a segmented truss design fabricated from SiC thin plates—a departure from the conventional use of metals or carbon fiber for such components. The secondary mirror assembly is also mounted via flexible hinges to the main support plate. The camera’s main system (comprising the primary mirror with backplate, the secondary mirror, and the primary support structure) was subjected to simulation analysis, followed by experimental testing and in-orbit imaging verification. Both the simulation results and test data satisfy the overall technical specifications for the space camera. The developed high-resolution camera thus meets the imaging requirements for microsatellite optoelectronic payloads.

## 2. General Requirements for Optoelectronic Payload (Space Camera) on Micro–Nano Satellite

The micro–nano satellite comprises several key subsystems: integrated electronics, attitude and orbit control, thermal management, and the space camera. Operating in a 500-km sun-synchronous orbit, it supports multiple imaging modes, including sun-pointing, scanning, and staring. The technical requirements imposed by the satellite system on its high-resolution space camera are summarized in Table 2.

## 3. Optical System Design

### 3.1. Coaxial Triple-Reflection Optical System

**Figure 1:**
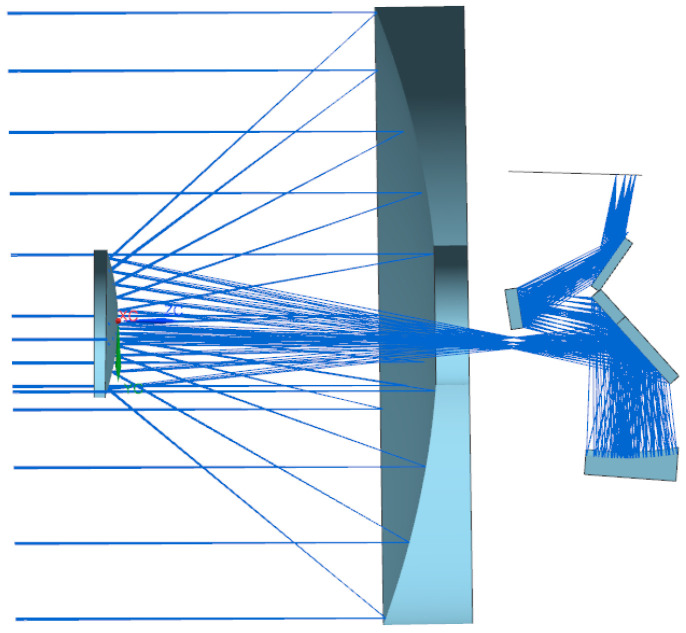
Schematic diagram of coaxial triple-reflection optical system.

To achieve both pushbroom and video imaging capabilities, the GMAX1205 CMOS sensor (Gpixel, Changchun, China) was selected as the camera’s image sensor. It features a pixel size of 2.8 μm and an effective pixel count of 7915 × 5436. The design aims for a swath width exceeding 6 km at a 500 km sun-synchronous orbit; therefore, two CMOS sensors were stitched together. A coaxial three-mirror anastigmatism (TMA) optical system was adopted. At a fixed focal length, a smaller relative aperture results in a lower diffraction limit and a smaller modulation transfer function (MTF) value at the Nyquist frequency. Additionally, the signal-to-noise ratio is proportional to the square of the relative aperture. To ensure sufficiently high diffraction limit and signal-to-noise ratio, the optical system design achieves an effective aperture of Φ500 mm and a resolution of 0.5 m at the subsolar point. Schematic diagram of coaxial triple-reflection optical system is illustrated in Figure 1. The primary mirror has a diameter of Φ510 mm, the secondary mirror is Φ125 mm, and the distance between them is 365 mm. By folding the optical path multiple times using mirrors, the overall camera length is significantly reduced from the original optical length of 800 mm to approximately 500 mm.

**Figure 2:**
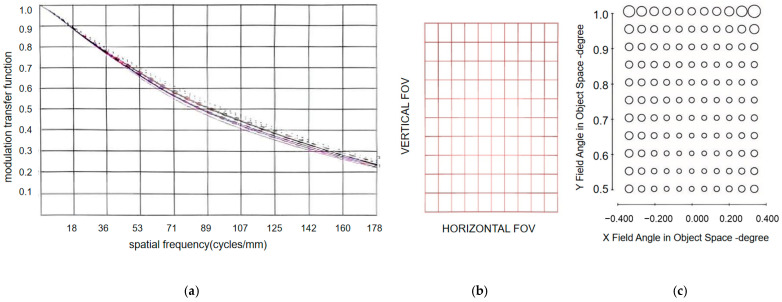
(**a**) MTF; (**b**) distortion map; (**c**) wave aberration.

The MTF of the coaxial triple-reflection optical system at the Nyquist frequency is shown in Figure 2a. The design transfer function curve of the optical system for reaching the diffraction limit is given, and the average modulation transfer function at the Nyquist frequency (178 PL/mm) reaches 0.24. Figure 2b indicates the optical system design transfer function curve, achieving the diffraction limit with distortion ≤ 0.69%; Figure 2c represents the optical system’s wavefront aberration, specifically the Strehl ratio, which exceeds 0.90 across all fields of view except the extreme periphery.

### 3.2. Tolerance Requirements for Optical Components

Tolerance analysis uses the primary mirror as the reference. The tolerances for the secondary mirror are analyzed with the design constraints set as a degradation of no more than 0.01 in the modulation transfer function (MTF) at the center field and 0.012 at the edge field, evaluated at the optical system’s Nyquist frequency. The camera uses the primary mirror backing plate as its main support structure. Among the support structure components for each mirror, both the primary and secondary mirrors employ flexible supports. This design introduces local compliance to compensate for manufacturing and alignment errors on the high-precision optical surfaces. It also eliminates the influence of temperature variations and differing coefficients of thermal expansion on the surface accuracy of the mirrors. The overall assembly must maintain high specific stiffness and strength. With the primary mirror as the reference, the permissible manufacturing and alignment tolerances are listed in Table 3.

## 4. Mechanical and Thermal Control System Design

**Figure 3:**
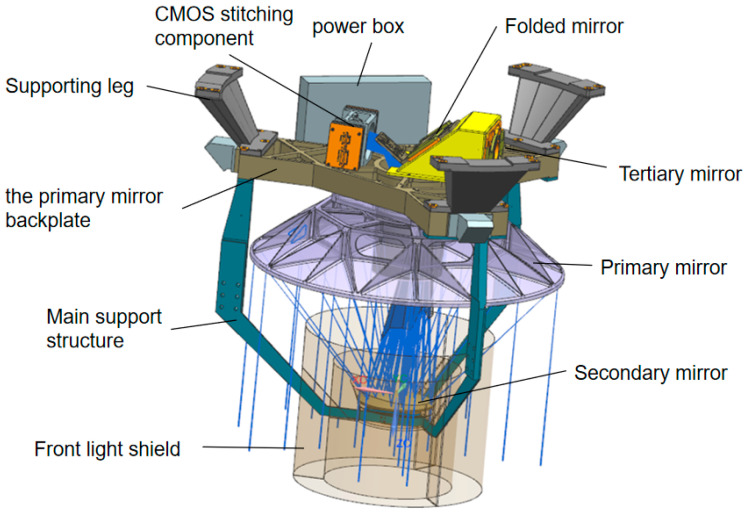
The overall model of the space camera.

The camera’s opto-mechanical structure is fundamental to ensuring imaging quality [17,18]. The challenge lies in achieving high rigidity, high mechanical and thermal stability, and low weight for wide-field space cameras. This ensures that the mirror surface figure and relative positions remain within optical design tolerances in the space environment, enabling the entire system to deliver excellent imaging performance during on-orbit operation. An overall assembly drawing of the space camera is provided in Figure 3.

The camera primarily consists of three major assemblies: the primary mirror assembly, the secondary mirror assembly, and the main support structure. Additional components include the aperture stop assembly, CMOS assembly, tertiary mirror and fold mirror assemblies, and the power unit. The primary mirror assembly is the heaviest component of the camera, mounted to the primary mirror backplate via flexible hinges. The CMOS assembly, tertiary mirror assembly, fold mirror assembly, and power box are all mounted on the backplate, which is integrated with the primary mirror backplate. The secondary mirror is fixed to the secondary mirror mount via adjustment shims. The main support is fixed to the secondary mirror mount via a flexible hinge. The main support structure integrates the camera into a unified assembly, which connects to the satellite platform via three support legs. This compact design ensures minimal volume and mass while maintaining exceptional image quality under external loads. It maximizes space utilization by minimizing the distance between the focal plane assembly and the CMOS readout circuitry, thereby enhancing the image signal-to-noise ratio. Additionally, positioning the components closer to the satellite platform facilitates efficient image data transmission. All camera mirrors, the main support structure, and the primary mirror backplate are fabricated from silicon carbide (SiC). This uniform material selection enables an athermalized camera design, significantly enhancing the system’s environmental stability. The development of the SiC main support structure—a pioneering application in this field—notably increases the overall fundamental frequency and substantially reduces the risk of resonance during launch.

The CMOS assembly consists of two tiled detector panels. After light is split by a prism, it forms images on each CMOS panel, which are then stitched together digitally. This approach expands the camera’s swath width and effectively shortens the revisit cycle for the microsatellite.

### 4.1. Thermal Environment Analysis

During in-orbit operation, the camera encounters a complex space environment. For analytical convenience, potential extreme operating conditions are categorized into low-temperature and high-temperature scenarios. The low-temperature scenario corresponds to the satellite’s solar panel Z-axis full sun-tracking mode, where the camera receives no external heat flux, ceases imaging operations, and maintains its surface state as at launch. The high-temperature condition corresponds to the camera’s +Z-axis Earth-pointing mode, where external heat flux reaches its maximum value. The camera performs imaging operations, and its surface state resembles the late launch phase. According to the satellite’s overall requirements, during in-orbit operation, the camera points toward Earth along the +Z-axis, receiving solar radiation, Earth infrared radiation, and regional albedo external heat flux. Directly exposed to the space environment, the camera experiences alternating heat flux variations, resulting in non-uniform temperature distribution. The camera will experience asymmetric thermal variations, necessitating stable temperature control during in-orbit operation. Addressing these operational characteristics and technical challenges, the overall thermal control design adopts a primary passive thermal control approach supplemented by active thermal control. This integrated strategy ensures the camera’s temperature meets specified requirements.

During on-orbit operation, the camera is exposed to a complex space environment. For analytical convenience, potential extreme thermal cases are categorized into cold and hot scenarios. The cold case corresponds to the satellite’s solar panel Z-axis sun-tracking mode, where the camera receives minimal external heat flux, does not perform imaging, and remains in a standby thermal state similar to that during launch. The hot case corresponds to the camera’s +Z-axis nadir-pointing mode, where the incident heat flux reaches its maximum. In this mode, the camera performs imaging operations, and its external thermal environment resembles that of the late launch phase. According to the satellite’s overall requirements, during nominal on-orbit operation with the camera in nadir-pointing (+Z-axis) mode, it is subjected to external heat fluxes from solar radiation, Earth infrared radiation, and Earth albedo. Directly exposed to the space environment, the camera experiences cyclic variations in heat flux, resulting in a non-uniform temperature distribution and asymmetric thermal gradients. Therefore, precise temperature control is required to maintain stability during operation. To address these operational characteristics and technical challenges, the thermal control design primarily employs a passive approach, supplemented by active measures. This integrated strategy ensures the camera’s temperature remains within the specified range.

**Figure 4:**
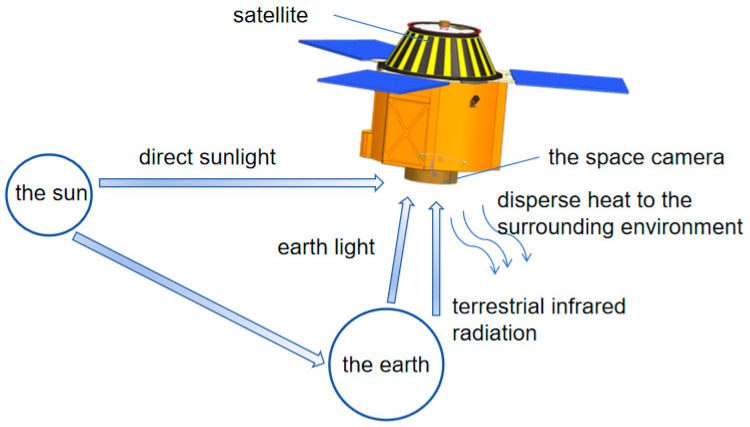
Exo-thermal flow around space cameras.

External heat fluxes around space cameras primarily consist of solar radiation, Earth’s infrared radiation, Earth’s albedo, and inter-component radiative exchange, as illustrated in Figure 4. During on-orbit operation, the camera is subjected to solar radiation. Calculations use an average solar constant of S = 1364 W/m^2^, with values of S = 1324 W/m^2^ at the summer solstice and S = 1414 W/m^2^ at the winter solstice [19]. Earth’s albedo is characterized by the Earth’s albedo coefficient. Due to the spacecraft’s high orbital velocity and the Earth’s large thermal inertia, its quasi-steady thermal state changes slowly relative to the orbital period. Therefore, a global average albedo coefficient (α) of 0.30–0.35 is used in analysis and calculations. Earth’s infrared radiation spans wavelengths from 2 to 50 μm. For spacecraft thermal analysis, the Earth can be approximated as a blackbody at approximately 250 K. In thermal design, Earth’s infrared radiation flux can be calculated using Equation (1):

(1) $$E_{io}={\left(1-\alpha \right)}^{4}S$$

Using Formula (1), it is calculated that $E_{io}$ varies between 236.3 W/m^2^ and 252.4 W/m^2^.

### 4.2. Main Support Structure Design

The main support structure of a space camera is one of its critical components. Optical elements (primary and secondary mirrors) are mounted on this structure. To meet high-resolution optical imaging quality requirements, the design must ensure the surface accuracy of each mirror and its relative positional accuracy. Additionally, it must withstand the immense loads during launch and the harsh conditions of space. The challenge lies in rationally designing the main support structure so that, after undergoing vibration, thermal loads, and gravity release, the changes in the surface accuracy and positional accuracy of each mirror remain within tolerance limits while achieving the lightest possible mass. Due to the large spacing between the primary and secondary mirrors in the space camera optical system, the requirements for relative positional accuracy and stability are extremely high, making the design highly challenging.

Current main support structures typically employ cylindrical or truss configurations. For instance, the main support structures (MSS) of space cameras on Quickbird-2, KompSat-3, GeoEye-1, Formosat-2, and ALSAT-2A are all of the cylindrical type [20,21,22]. Truss designs [23,24,25,26] include carbon fiber truss and cast aluminum variants. Cylindrical and carbon fiber truss structures feature larger overall dimensions, while cast aluminum alloy structures are heavier and typically suited for smaller optical payloads. Existing research on support structures primarily focuses on traditional materials such as aluminum alloys, carbon fiber, and titanium alloys. The potential of silicon carbide materials in main support systems remains to be explored, as the application of novel materials can significantly enhance the thermal stability and lightweight performance of these systems.

Through comparative design analysis, a thin-plate main support structure made of RB-SiC material was found to simultaneously meet the comprehensive requirements of high fundamental frequency, light mass, and compact volume. The fundamental frequency critically influences the overall system stiffness. Therefore, the support structure underwent topology optimization with the fundamental frequency as the objective to determine the optimal load path. Following topology optimization, an initial structural design was developed by retaining areas of higher material density based on the optimization results. Subsequently, parameter optimization was performed with the objective of maximizing the first-order natural frequency to determine the final configuration of the main support structure.

#### 4.2.1. Topology Optimization Theory

The Solid Isotropic Microstructure with Penalization (SIMP) method [14] is a commonly used approach in continuum topology optimization. Based on the variable density method, topology optimization of the main support structure is performed with the objective of identifying the optimal load path. Using the relative density of each element as the design variable, the material interpolation scheme relating the density to the elastic modulus is described by the following constitutive relation:

(2) $$E=\rho^{p}E_{0}$$

In the equation, E represents the material’s elastic modulus; ρ denotes the material density; $E_{0}$ signifies the initial value of the material’s elastic modulus; p is the penalty factor (set to 3 for solid elements), enabling the element density to seek an optimal distribution between 0 and 1.

Variable density topology optimization methods have been widely applied in lightweight structural design for space cameras. Given the extremely harsh environments in which cameras operate, high fundamental frequencies are required to ensure superior stability. The topology design is conducted with the objective of maximizing the first-order natural frequency to determine optimal material distribution. To ensure the support structure possesses high static stiffness under gravity, mass points representing the primary and secondary mirror assemblies are used to apply gravitational loads. The optimization constraint requires rigid-body displacement of both mirrors to remain below 10 µm. To reduce the support mass and improve optimization efficiency, a 35% upper limit on volume fraction was imposed. Assuming the support structure to be a single-degree-of-freedom, undamped system whose characteristic frequency is the design objective, the mathematical model for its topology optimization is defined as follows:

(3) $$\begin{array}{l} \mathrm{find}\;X={\left(\rho_{1},\;\rho_{2},\ldots ,\;\rho_{n}\right)}^{T}\\ \max\;(\omega_{1}^{2})\\ s.t.\;K\varphi_{1}-\omega_{1}^{2}M\varphi_{1}=0\\ \;\;\varphi_{1}^{T}M\varphi_{1}-1=0\\ \;\sum_{i=1}^{n}\rho_{i}V_{i}-\alpha V^{0}\leq 0\\ \;\mathrm{Max}\left(d_{1},d_{2}\right)<10\mathrm{um}\\ \;0<\rho_{\min}<\rho_{i}\leq 1\left(i=1,\;2,\;\ldots ,\;n\right) \end{array}$$

In the equation, $\rho_{i}$ (i = 1, 2, …, N) represents the density of element i, which is the product of the material density and the penalty factor; $\omega_{1}$ is the first-order characteristic frequency; K and M denote the stiffness matrix and mass matrix, respectively; $V_{i}$ denotes the volume of element i; $V^{0}$ is the volume of the design domain; α is the volume fraction of the design domain. n is the total number of elements; $\varphi_{1}$ is the first-order characteristic vector; $d_{1}$ and $d_{2}$ represent the maximum rigid-body displacements of the two mirror assemblies under gravitational loading; $\rho_{\min}$ is the minimum element density, set to $\rho_{\min}$ ≥ 1 × 10^−3^ to prevent matrix singularity.

#### 4.2.2. Theory of Dimensional Parameter Optimization

Following topology optimization, the optimal material distribution for the support structure was obtained. However, the resulting topology is not directly manufacturable and requires design interpretation to create an initial structural design compliant with available manufacturing processes. Since the initial structural dimensions are not yet optimized, further parametric optimization is necessary. The objective for the primary support structure is to maximize the first-order natural frequency, subject to a constraint that the fundamental frequency exceeds a specified minimum. Additionally, all dimensional parameters are constrained to lie within maximum and minimum values. The mathematical model for this dimensional parameter optimization is established as follows:

(4) $$\begin{array}{l} \max\;f_{1}\\ s.t.\\ \;\;f_{1}\geq f_{0}\\ L_{\min}\leq \left(L_{e}\right)\leq L_{\max},\;e=1,2,\ldots ,n \end{array}$$

In the equation, $f_{1}$ is the first natural frequency; $f_{0}$ is the minimum frequency; $L_{\min}$ is the minimum value of the dimension; $L_{\max}$ is the maximum value of the dimension; $L_{e}$ is the dimensional variable. n is the total number of dimensions requiring optimization.

**Figure 5:**
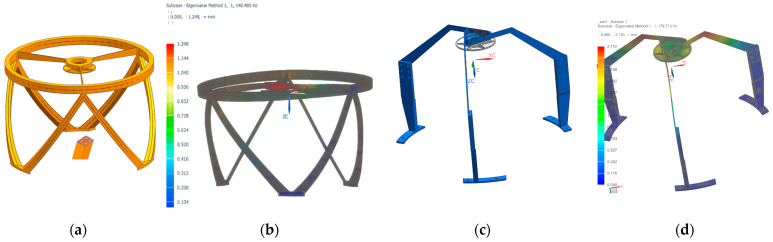
(**a**) Main support solution model type 1; (**b**) the first modal shape of type 1; (**c**) main support solution model type; (**d**) the first modal shape of type 2.

Two primary support design concepts were proposed: one utilizing carbon fiber material, which has a lower density than silicon carbide and offers good toughness but provides lower stiffness; the other employing silicon carbide material. Based on theoretical Equations (3) and (4), the optimized design yielded the final main support structure models shown in Figure 5a,c. The total mass of the carbon fiber truss assembly is 1.321 kg, while the silicon carbide truss has a mass of 1.411 kg. Modal analysis was performed on both structures, with results shown in Figure 5b,d. With the secondary mirror installed, their fundamental frequencies are 140.48 Hz and 179.71 Hz, respectively. Consequently, the main support structure made of silicon carbide exhibits superior resistance to vibration-induced damage. Since both mirrors are fabricated from silicon carbide, thermal deformation due to coefficient of thermal expansion mismatch is eliminated, enabling better preservation of mirror surface accuracy. With a thickness of only 3 mm, the silicon carbide support structure minimizes obscuration of imaging light rays. The carbon fiber truss, featuring a cross-hollow structure and curved surfaces at its center as shown in Figure 5a, presents greater manufacturing complexity and requires a longer development cycle. In contrast, the silicon carbide primary support structure is simpler and easier to fabricate. After comprehensive consideration, the silicon carbide material was ultimately selected for the main support structure.

No published reports exist on silicon carbide primary support structures, leaving no established design precedents to follow. Given the challenges associated with machining silicon carbide, the support structure employs a segmented design comprising a support base, connecting plate, secondary mirror mount, and flexible hinge. The secondary mirror is mounted using screws and precision shims, while the distance and positional accuracy between the primary and secondary mirrors are adjusted by precision grinding of the shims. The secondary mirror mount is connected to the flexible hinge via screws, while the flexible hinge is bolted to the connecting plate. This configuration establishes a flexible connection between the secondary mirror and the connecting plate, which helps isolate the mirror from external loads, preserving its surface accuracy and reducing its sensitivity to environmental variations.

### 4.3. Reflector Structure Design

The mirror support structure components for the 0.5 m microsatellite camera are designed with strategically placed flexible elements. These elements introduce local compliance, mitigating manufacturing and assembly errors and decoupling the optical surfaces from the effects of temperature variations and differences in coefficients of thermal expansion. Each component is designed to maintain high specific stiffness and stability. This flexible design meets strength requirements while preventing failure or micro-yielding under severe shock and vibration. As a critical element in the camera’s optical system, the primary mirror’s support method, assembly, and alignment accuracy directly affect its surface figure and, consequently, the camera’s imaging quality [27,28,29]. To ensure this quality, all mirror assemblies (e.g., primary and secondary) must possess excellent static/dynamic mechanical and thermal properties to maintain stability under varying environmental conditions.

#### 4.3.1. Design Theory for Reflector Structures

Optimization of mirror designs typically employs empirical methods, topology optimization, and parametric optimization. Initial designs for mirror support structures, lightweight elements, and contour shapes are developed based on existing design experience. The primary mirror’s thickness and number of support points were determined using classical empirical Formulas (5) and (6) [30,31], which established the initial structure of the primary mirror.

(5) $$\begin{array}{l} \delta =\frac{3\rho gr^{4}}{16Et^{2}}=\frac{3\rho g{\left(\partial \right)}^{2}D^{2}}{256E} \end{array}$$

(6) $$N=\left(\frac{1.5r^{2}}{t}\right)\sqrt{\frac{\rho g}{E\delta }}$$

In Equations, N is the smallest number of rear support points. r is the radiator radius. δ is the maximum deflection of the surface under its own weight. ρ is the material density. E is the elastic modulus of the primary mirror material. t is the height, g is the gravitational acceleration. δ is the value of the PV for the surface shape accuracy. ∂ is the ratio of diameter to thickness.

Based on design experience, an initial structural design for the mirrors is established, followed by dimensional parameter optimization to achieve the optimal structural configuration. During dimensional parameter optimization, the objectives are set as minimizing the mirror mass and surface deformation. Constraints include the requirement that the fundamental frequency exceeds 200 Hz, a uniform temperature of 20 ± 4 °C, and that all optimized dimensions remain within predetermined maximum and minimum values. A mathematical optimization model for the mirrors has been established, as shown in Equation (7). The mirrors’ dimensions were optimized using Equation (7) to achieve the optimal mirror structure.

(7) $$\begin{array}{l} {\mathrm{find}:\;X=(H}_{1},H_{2},H_{3},\ldots ,H_{n})\\ \min:(RMS,Mass)\\ s.t.\;f_{1}\geq f_{0}=200\mathrm{Hz}\\ 20\;℃-4\;℃\leq T\leq 20\;℃+4\;℃\\ \;\;H_{\min}\leq \left(H_{e}\right)\leq H_{\max},\;e=1,2,\ldots ,n \end{array}$$

In Equation (7), $H_{e}$ is the size optimization variable; Mass is the final mass of the reflector; $f_{1}$ is the first-order frequency of the reflector; $f_{0}$ is the minimum frequency for the reflector; T is the ambient temperature; $H_{\min}$ is the minimum value of the optimization variable; $H_{\max}$ is the maximum value of the optimization variable.

#### 4.3.2. Primary Mirror Structural Optimization Design

**Figure 6:**
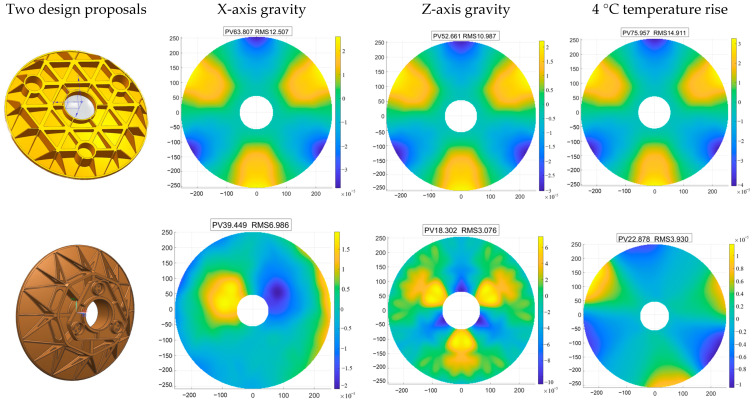
Results of comparative analysis of two support schemes for the primary mirror.

According to the optical system requirements, the primary mirror has an effective aperture of 500 mm. To prevent chipping during machining, the diameter was increased by 10 mm, yielding a final primary mirror diameter of 510 mm with a center bore of 116 mm. The reflective surface is aspherical and fabricated from silicon carbide material. The primary mirror’s initial structure was determined based on design experience regarding mirror thickness and support point count. Two design approaches were evaluated. The first employs a conventional three-point support method at the rear, featuring tapered sleeves and flexible hinges at the support points [32,33,34]. The second adopts an integrated structure where the mirror body and support plate are unified. The three support points on the plate are secured to the backplate via screws. Finite element simulation analysis was conducted, with comparative results shown in Figure 6. Analysis reveals that under X-axis gravitational loading, both designs exhibit minimal surface accuracy deviation. However, under Z-axis gravitational loading, significant surface accuracy differences are observed, with the traditional support structure demonstrating greater sensitivity to temperature variations. This paper adopts the second approach. This integrated design alters the load path, directing stresses directly to the support plate. The flexible hinge releases partial internal stresses, thereby reducing the surface accuracy’s sensitivity to temperature. In the traditional design, support forces act directly on the mirror surface, causing deformation under external loads. Following initial structural design, key dimensions of the primary mirror were optimized using mathematical model (7). The final design achieved an 85% weight reduction, with a total mass of 4.57 kg, meeting the satellite’s overall mass specification of not exceeding 5 kg.

#### 4.3.3. Primary Mirror Thermal Control Design

**Figure 7:**
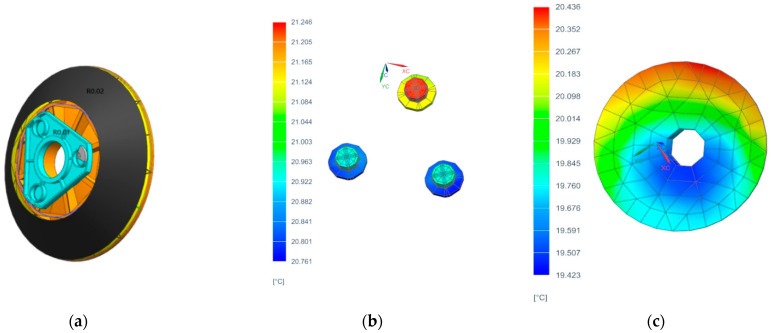
(**a**) Thermal control system of the primary mirror.; (**b**) thermal analysis contour map of the primary mirror’s flexible hinge; (**c**) thermal analysis contour map of the primary mirror.

Under low-temperature conditions, the external heat flux scenario for the satellite solar panel’s +Z-axis sun-tracking mode is set to the minimum external heat flux, corresponding to the summer solstice with a solar constant of 1324 W/m^2^. The thermal state of the camera’s multilayer insulation corresponds to its initial launch configuration, and the satellite cabin temperature is −20 °C. The thermal control design for the primary mirror assembly is shown in Figure 7a. The heater is mounted on the mirror body. Uneven temperature distribution across the primary mirror assembly can cause thermal deformation of the mirror surface. Therefore, an auxiliary heating shroud is installed on the rear surface of the primary mirror. The auxiliary heating shroud is divided into two heating zones: the primary mirror connection plate zone, designated as R0.01, with a power consumption of 14 W, and the auxiliary heating shroud zone, designated as R0.02, with a power consumption of 4 W. A steady-state thermal analysis of the primary mirror assembly was conducted based on the camera’s thermal control conditions, yielding steady-state temperature distributions during operation (Figure 7b,c). The figure shows that at steady state, the temperature of the primary mirror assembly remains within the range of 20 ± 4 °C, meeting the thermal control requirements.

#### 4.3.4. Secondary Mirror Structure and Thermal Control Design

**Figure 8:**
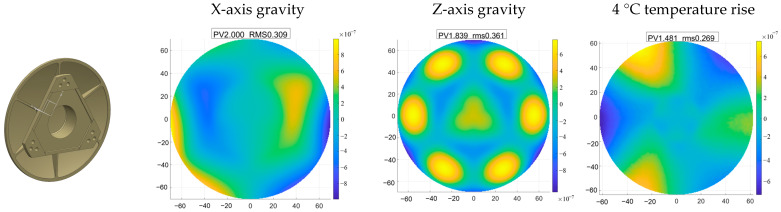
Simulation results of the secondary mirror.

The secondary mirror has a diameter of 125 mm with an aspherical surface, and the material selected is silicon carbide. Due to its small aperture, the silicon carbide secondary mirror typically adopts a center-supported design, with the support force directly applied to the mirror body. This paper employs a design where the secondary mirror is fixed to its mount with screws. A flexible hinge is anchored at one end to the secondary mirror mount and at the other end to the main support structure. This arrangement allows for independent, flexible adjustment of the secondary mirror, effectively achieving flexible adjustment of the entire secondary mirror assembly. This significantly reduces the sensitivity of surface accuracy to external loads, particularly thermal loads. Finite element analysis results, as shown in Figure 8, demonstrate a surface accuracy RMS significantly below the overall specification requirement of no more than λ/60.

**Figure 9:**
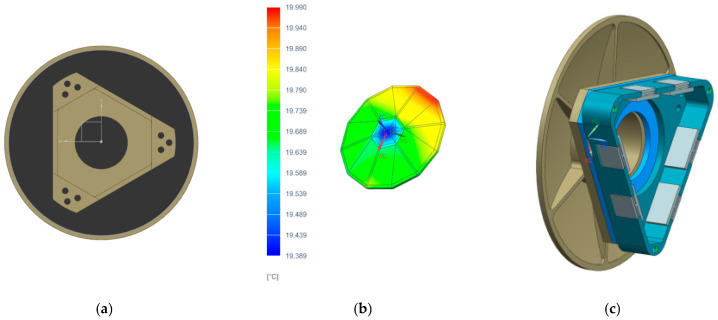
(**a**) thermal control system of the secondary mirror; (**b**) thermal analysis contour map of the secondary mirror; (**c**) secondary mirror assembly.

A radiator/heater assembly is installed on the outer side of the secondary mirror backplate, designated as heating zone R0.03 with a power consumption of 3 W. The secondary mirror thermal control system is shown in Figure 9a. A steady-state thermal analysis was conducted for the secondary mirror assembly based on the thermal control conditions. The resulting temperature distribution is shown in Figure 9b. The figure indicates that at steady state, the assembly’s temperature remains within the range of 20 ± 4 °C, meeting the thermal control requirements and the assembly of the secondary mirror and its flexible support is shown in Figure 9c.

## 5. Engineering Analysis

**Figure 10:**
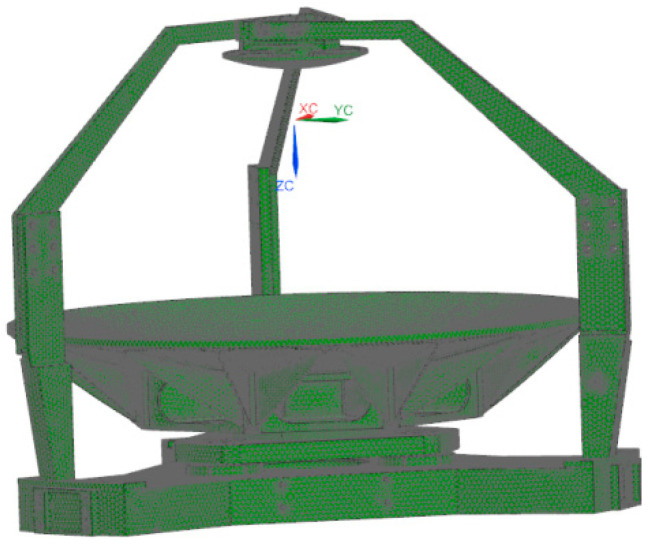
The finite element mesh model of the assembly.

The primary structure consists of the primary mirror assembly, secondary mirror assembly, and main support structure. After meshing the structural system, assigning materials, and defining bonded contacts, finite element analysis was conducted. The mesh model is shown in Figure 10. The model materials are assigned as follows: the primary mirror and secondary mirror are made of silicon carbide, while the primary mirror flexible hinge, secondary mirror flexible hinge, and secondary mirror adjustment shims are made of 4J32 alloy. The material properties are shown in Table 4.

### 5.1. Static Analysis

To validate the design, a static analysis was first performed on the entire system. Both the displacement and tilt were analyzed relative to the primary mirror. Under the coupled effects of gravity, a uniform temperature increase of 4 °C, and an assembly flatness error of 0.01 mm, the surface figure errors (RMS), displacements, and tilt angles of the primary and secondary mirrors are summarized in Table 3. The RMS surface error of both mirrors, the relative displacement of the secondary mirror, and their tilt angles all meet the specified requirements in Table 5.

### 5.2. Modal Analysis

Modal analysis is applied to determine the dynamic characteristics of opto-mechanical structures. By solving the eigenvalue problem of the structure for its mode shapes and natural frequencies, it provides a theoretical basis for optimizing the structure’s dynamic performance. The natural frequencies and mode shapes of a camera’s opto-mechanical system are inherent properties, determined by its constraints, materials, and geometry, and are independent of external loads. To evaluate the dynamic performance of an opto-mechanical system, modal analysis must be conducted to avoid resonance between the system’s fundamental frequency and the excitation frequencies during rocket launch, which could cause damage to the structure.

**Figure 11:**
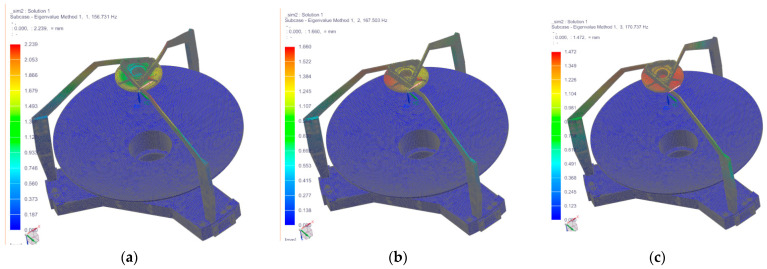
The results of the first three natural modes of vibration analysis. (**a**–**c**) the first to third vibration shape diagram.

The high natural frequency of the opto-mechanical system indicates high dynamic stiffness. Performing modal analysis of the opto-mechanical system through finite element analysis is crucial for verifying the safety of its structural dynamic stiffness. Typically, the first-order frequency of the opto-mechanical system should be at least twice the excitation frequency generated during launch to prevent resonance. Finite element analysis was employed to evaluate whether the primary support structure possesses sufficient stiffness and stability. This ensures the relative position accuracy of the camera mirror assembly remains within tolerance limits, thereby guaranteeing imaging quality. The dynamic analysis of the entire system primarily involves modal analysis of the primary mirror assembly, secondary mirror assembly, and their supporting structures. Modal simulation was conducted with the lower end of the backplate as a fixed constraint. The first three natural frequencies and their corresponding mode shapes are shown in Figure 11. The first natural frequency is 156.731 Hz, with rotation about the Z-axis. The second natural frequency is 167.503 Hz, with translation along the X-axis. The third natural frequency is 170.737 Hz, with translation along the Y-axis. The fundamental frequency meets and exceeds the satellite’s requirement of 100 Hz.

## 6. Experimental Verification

### 6.1. Wavefront Aberration Detection

**Figure 12:**
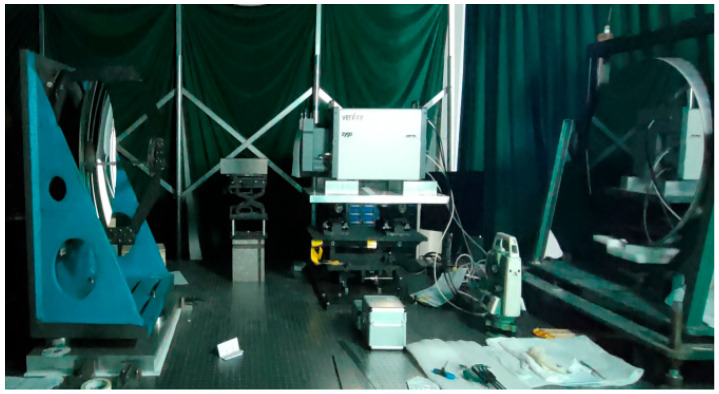
Wavefront aberration measurement setup.

**Figure 13:**
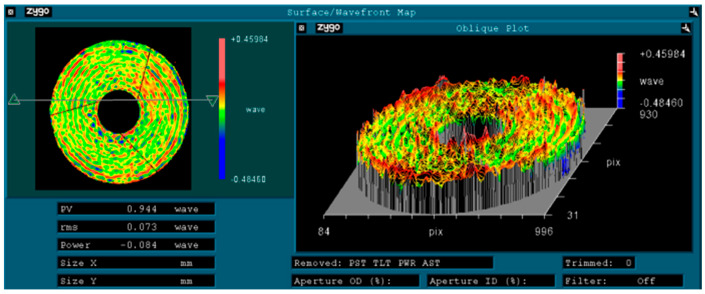
Wavefront aberration detection result.

The primary mirror, secondary mirror, and primary support truss were developed using the RB-SiC reaction sintering method and an integrated manufacturing process. Threaded connections were employed between the primary mirror’s flexible support and the connecting plate, between the connecting plate and the primary mirror backing plate, between the secondary mirror assembly and the main support structure, between the primary mirror backing plate and the primary support baseplate, and between the secondary mirror mount and the secondary mirror flexible hinge. The primary mirror was installed first. Subsequently, using the primary mirror as a reference, the secondary mirror’s position relative to the primary mirror was precisely adjusted by grinding the adjustment shims. The space camera employs a coaxial triple-mirror system. To meet the system’s imaging quality requirements, the relative positional accuracy between the mirrors must first be ensured. Each mirror possesses six degrees of freedom, totaling twelve degrees of freedom for the two mirrors. During system assembly and calibration, the primary mirror is first fixed. The secondary mirror, with its rotationally symmetric structure, allows one rotational degree of freedom (about the Z-axis) to be ignored, leaving five degrees of freedom to be adjusted. The position of the secondary mirror is refined by grinding adjustment pads to achieve optimal system imaging. During the gravity test, the camera’s optical axis was maintained in a horizontal state, and wavefront error tests were conducted under gravitational conditions. On-site photographs of the wavefront error measurement are shown in Figure 12. The wavefront interferogram is shown in Figure 13. Under gravitational influence, the RMS value of the system wavefront error in the central field of view was 0.073λ, which meets the overall requirement of ≤1/14λ, satisfying the overall technical specifications. This indicates that the camera exhibits excellent static performance and stability.

### 6.2. Mechanical Vibration Test

**Figure 14:**
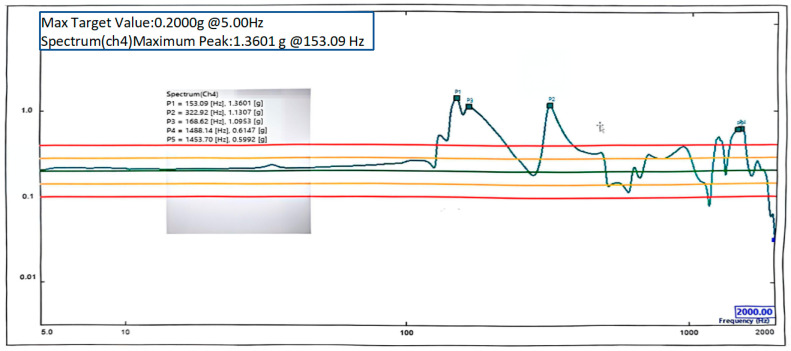
The Z-direction vibration result.

First, the primary mirror was mounted to the backplate. Then, the primary support structure was installed. Finally, the secondary mirror and shims were adjusted to achieve the specified system wavefront error requirements, and then the secondary mirror was secured. The primary support structure is a cantilevered structure, which may exhibit dynamic amplification during mechanical testing under simulated launch conditions. To validate the feasibility and reliability of the structural design, environmental testing was conducted. These tests effectively simulate the mechanical environment experienced by the structure, evaluate interactions between components, and release assembly stresses. Sine vibration tests were conducted at an acceleration level of 0.2 g. Since modal simulation analysis indicated the fundamental frequency is associated with motion in the Z-axis, vibration testing was performed along the Z-axis. Sensors were mounted on the main support structure to obtain accurate feedback signals. Test results are shown in Figure 14. The results indicate that the first-order natural frequency of 153.09 Hz significantly exceeds the specified requirement of 100 Hz from Table 2 and closely aligns with the simulated fundamental frequency of 156.731 Hz. This confirms the accuracy of the simulation model and demonstrates that the structure possesses sufficient stiffness and stability.

### 6.3. In-Orbit Imaging

**Figure 15:**
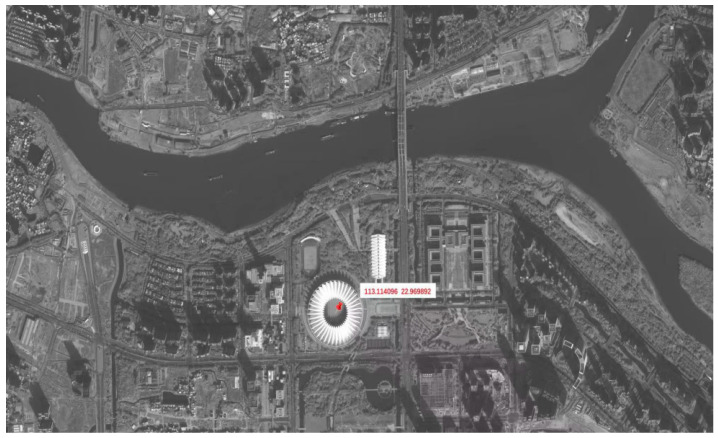
In-orbit imaging satellite photograph.

The space camera utilizes a design in which the imaging light path is split and directed onto two tiled CMOS detectors. The two individual sub-images are then digitally stitched. Following on-ground processing of the on-orbit images, this method achieves wide-swath imaging. The downlinked satellite image, as shown in Figure 15, exhibits high resolution and excellent image quality with no visible stitching artifacts, meeting the design objectives.

## 7. Conclusions

To meet the overall requirements for high-resolution electro-optical payloads on microsatellites, such as achieving 0.5-m ground resolution from a 500-km orbit with a total mass under 50 kg, an opto-thermo-mechanical system of the space camera was designed and experimentally validated. First, an ultra-compact coaxial three-mirror optical system was designed. By minimizing the F-number to maximize light throughput, the optical parameters were determined. Subsequently, tolerance ranges for the primary and secondary mirrors—including surface accuracy, dimensional tolerances, and positional tolerances—were established based on the optical design. Mathematical models for topology optimization and parametric optimization were developed. A comparative optimization study and finite element simulation were conducted for the main support structure and the primary mirror assembly. Simulation of the main system confirmed that all performance metrics met the overall requirements for the space camera. The simulated fundamental frequency of the opto-mechanical system was 156.731 Hz, significantly exceeding the satellite requirement of 100 Hz. Subsequently, the primary mirror assembly, secondary mirror assembly, and primary support structure were precision-machined and assembled based on the final solid model. The assembled system then underwent wavefront error testing, vacuum optical performance testing, vibration testing, and in-orbit imaging verification. Test results indicate a system wavefront error of 0.073λ (λ = 632.8 nm), meeting the technical requirement of ≤λ/14. The measured fundamental frequency is 153.09 Hz, closely matching the simulated value. The total camera mass is under 30 kg, well below the 50 kg requirement. The use of silicon carbide (SiC) for all optical components and the main support structure significantly enhances the camera’s thermal stability, overall stiffness, and lightweight characteristics. This work provides new approaches and methodologies for designing opto-thermo-mechanical systems for high-resolution microsatellite cameras.

## Figures and Tables

**Table 1:** Microsatellites with sub-meter ground resolution.

State	MicroSatellite	Ground Resolution (m)	Weight (kg)
Argentina	NewSat series satellites	1	40
United States	SkySat satellite	0.5	100
China	“Jilin-1” Gaofen-03 Series Satellite	0.75	40
United States	BlackSky Series Satellites	0.9	44
Finland	ICEYE Synthetic Aperture Radar Satellite	0.5	85

**Table 2:** Overall technical specifications of the camera.

Components	Value
Orbital height	500 km
ground resolution	0.5 m
System wavefront aberration	RMS≤λ/14
Focal length of the optical system	2800 mm
Spectral range	450 nm~800 nm
ground-imaging swath width	≥6 km
Temperature control range	20 °C ± 4 °C
Maximum camera dimensions	Φ620 × 1140 mm
Fundamental frequency	≥100 Hz
Camera weight	≤50 Kg

**Table 3:** The permissible tolerances for the mirrors.

Components	Value
The Primary Mirror	PV≤λ/10;RMS≤λ/50;λ=632.8 nm
Secondary Mirror	PV≤λ/10;RMS≤λ/60
Secondary mirror translation along X-axis (DLX)	0.01 mm
Secondary mirror translation along Y-axis (DLY)	0.01 mm
Secondary mirror translation along Z-axis (DLZ)	0.06 mm
Secondary mirror tilt angle around the X-axis (DLA)	5″
Secondary mirror tilt angle around the Y-axis (DLB)	8″
Secondary mirror tilt angle around the Z-axis (DLB)	20″

**Table 4:** Material characteristics of the opto-mechanical systems.

Components	Material	Young’s Modulus (Gpa)	Poisson’s Ratio	Density (g/cm^3^)	Coefficient of Thermal Expansion (10^−6^/K)
Primary mirror	SiC	330	0.18	3.05	2.5
Flexure	4J32	141	0.25	8.1	2.5

**Table 5:** FEA results of coupling conditions.

Mirror	PV, RMS (nm)	DLX (μm)	DLY (μm)	DLZ (μm)	DLA″	DLB″	DLG″
Primary mirror	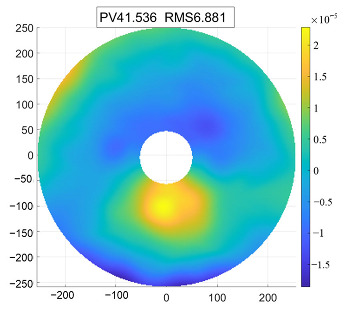	–	–	–	–	–	–
Secondary mirror	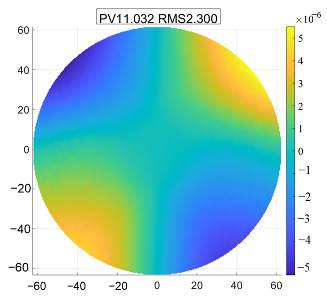	0.040	0.032	2.712	0.271	0.148	10.602

## Data Availability

The original contributions presented in this study are included in the article. Further inquiries can be directed to the corresponding authors.
